# Recombinant Filamentous Bacteriophages Encapsulated in Biodegradable Polymeric Microparticles for Stimulation of Innate and Adaptive Immune Responses

**DOI:** 10.3390/microorganisms8050650

**Published:** 2020-04-29

**Authors:** Rezvan Jamaledin, Rossella Sartorius, Concetta Di Natale, Raffaele Vecchione, Piergiuseppe De Berardinis, Paolo Antonio Netti

**Affiliations:** 1 Center for Advanced Biomaterials for Health Care (CABHC), Istituto Italiano di Tecnologia, 80125 Naples, Italy; Rezvan.jamaledin@iit.it (R.J.); Concetta.dinatale@iit.it (C.D.N.); paoloantonio.netti@unina.it (P.A.N.); 2 Institute of Biochemistry and Cell Biology (IBBC), CNR, 80131 Naples, Italy; rossella.sartorius@ibbc.cnr.it (R.S.); piergiuseppe.deberardinis@ibbc.cnr.it (P.D.B.); 3 Department of Chemical Materials and Industrial Production (DICMAPI), University of Naples Federico II, 80125 Naples, Italy; 4 Interdisciplinary Research Center of Biomaterials, CRIB, University Federico II, P.leTecchio 80, 80125 Naples, Italy

**Keywords:** vaccine delivery, bacteriophage display, microparticles, sustained-release, immunity

## Abstract

*Escherichia coli* filamentous bacteriophages (M13, f1, or fd) have attracted tremendous attention from vaccinologists as a promising immunogenic carrier and vaccine delivery vehicle with vast possible applications in the development of vaccines. The use of fd bacteriophage as an antigen delivery system is based on a modification of bacteriophage display technology. In particular, it is designed to express multiple copies of exogenous peptides (or polypeptides) covalently linked to viral capsid proteins. This study for the first time proposes the use of microparticles (MPs) made of poly (lactic-co-glycolic acid) (PLGA) to encapsulate fd bacteriophage. Bacteriophage–PLGA MPs were synthesized by a water in oil in water (w_1_/o/w_2_) emulsion technique, and their morphological properties were analyzed by confocal and scanning electron microscopy (SEM). Moreover, phage integrity, encapsulation efficiency, and release were investigated. Using recombinant bacteriophages expressing the ovalbumin (OVA) antigenic determinant, we demonstrated the immunogenicity of the encapsulated bacteriophage after being released by MPs. Our results reveal that encapsulated bacteriophages are stable and retain their immunogenic properties. Bacteriophage-encapsulated PLGA microparticles may thus represent an important tool for the development of different bacteriophage-based vaccine platforms.

## 1. Introduction

Bacteriophages are a powerful platform with outstanding potential in the biomedical and chemical engineering field that have been exploited for diverse applications including theranostics [1], batteries [2,3], drug delivery [4], and vaccine development [5]. Filamentous bacteriophages are single-strand DNA virions belonging to the Inoviridae family, a sub-group of non-lytic, rod-like shaped *Escherichia coli* viruses with a repeated and ordered capsid structure, and that includes phages f1, fd, and M13 [6]. Fd filamentous bacteriophage is a bio nano-fiber with a modifiable surface that is a promising vehicle for antigen expression. A considerable body of data has been accumulated concerning the molecular basis of structural and functional features of fd bacteriophage [7,8,9,10]. Fd bacteriophage genome is intrinsically rich in deoxycytidylate-phosphate-deoxy guanylate (CpG) regions, which can be recognized by toll-like receptors (TLRs). After activation of TLRs, signaling induces the generation of inflammatory signal mediators such as cytokines, and can develop adaptive immune responses without needing any exogenous adjuvant [11,12,13,14].

The major goal of vaccination is to induce a strong immune response and long-lasting immunity [15,16]. Due to the lack of an appropriate delivery system, some antigens are unable to induce a strong and effective immune response, and therefore the emergence of an optimal delivery system is of great interest for new vaccine formulations [17].

The fd filamentous bacteriophage antigen display system is a vaccine candidate that is able to induce both innate and adaptive immune responses [18,19,20,21]. Based on a modification of phage display technology, fd bacteriophage was engineered to express exogenous peptides in high copy numbers, as fusions to the N-terminus of viral capsid protein pVIII. Recombinant hybrid virions carry multiple copies of pVIII-containing exogenous sequences interspersed with wild-type pVIII copies on the phage coat. Peptide display on filamentous bacteriophages can be employed to present antigenic peptides to antigen presenting cells (APCs) and thus trigger a strong immune response. The implementation of phage-based vaccines can be reached by improving phage stability and obtaining well-defined pharmacokinetics and pharmacodynamics. Bacteriophages stored for long periods as solution can drop in phage titre, decreasing their therapeutic efficacy and the necessary systems for long-time preservation. In vivo injected bacteriophages distribute rapidly throughout the body, with most of the virions cleared from the bloodstream with a half-life of 4.5 h [22]. The loss of bacteriophage concentration in vivo may require repeated administrations, and encapsulations of bacteriophages may be used for a more prolonged release.

The development of genetically engineered bacteriophage preparations with improved pH resistance [23], embedded in hydrogel microspheres [24] or pH-responsive polymers [25] can ameliorate the stability of filamentous bacteriophage vaccines. Additionally, the encapsulation of virions into appropriate polymeric microparticles can improve the half-life of the bacteriophage by protecting it in harsh environments (e.g., the gastrointestinal tract at low pH, if administered orally). Furthermore, the encapsulation in a biodegradable porous system can modulate the release of bacteriophages, leading to a prolonged stimulation of the immune system over time and increasing the immunogenicity of the phage-based vaccine [26,27]. In this work, PLGA was used as a biodegradable and biocompatible polymer to fabricate microparticles (MPs) through the water in oil in water (W_1_/O/W_2_) double-emulsion/solvent evaporation method [28,29]. However, for several proteins, it has been shown that the presence of a large interface in double emulsion between aqueous and organic solvents such as dichloromethane (DCM) is responsible for protein degradation during emulsification. Furthermore, there are some mechanical stresses applied during fabrication, including shear stress during mixing and agitation [19,26]. For this reason, we decided to fabricate empty PLGA microparticles then load bacteriophages into their open porosities, in order to avoid antigen degradation and preserve an intact bacteriophage structure. Our dry formulations of phage MPs may represent a promising alternative to common bacteriophage-based therapeutic administrations and offer advantages to the field of vaccination, including longer phage stability, better antigen delivery, prolonged vaccine release and improved shelf life.

## 2. Materials and Methods

### 2.1. Bacteriophage Purification

Recombinant hybrid bacteriophage fdOVA (expressing ovalbumin peptide SIINFEKL (residues 257–264)-pVIII proteins) was generated as described elsewhere [30]. Briefly, DNA oligos encoding the OVA (257–264) MHC H-2^b^-restricted peptide (5′-CCGCGGAGGGTTCCATCATCAACTTCGAAAAACTGGACGATCCCGCCAAGG-3′) were cloned into SacII-StyI-digested fdAMPLAY88 phage genome [31] containing two copies of pVIII proteins: one wild-type and one with the SacII-StyI restriction sites. This second copy was under the control of the isopropyl-beta–d-thiogalactopyranoside (IPTG)-inducible promoter pTac. *E. coli* TG1*recO* cells, transformed with recombinant bacteriophages fdOVA DNA, produced hybrid phages in the supernatant. Wild-type fd and hybrid fdOVA filamentous bacteriophages were purified from the supernatant of *E. coli* TG1*recO* cells previously transformed with the phage DNA, according to [32].

Briefly, bacteria transformed with the phage DNA were grown in 2XTY medium (16 g/L Tryptone, 10 g/L Yeast Extract, 5.0 g/L NaCl) for 16 h in the presence of 100μg/mL Ampicillin. The expression of the recombinant OVA-pVIII proteins was induced by adding 0.1 mM IPTG (Sigma-Aldrich, Milan, Italy) to the growing cultures (at Absorbance A_600_ = 0.25 optical density (OD)). The supernatant containing bacteriophages was harvested from *E. coli* cultures by centrifugation, and phages were subjected to double precipitation by adding 20% polyethylene glycol 6000 (PEG, Sigma-Aldrich) and 2.5M NaCl (Sigma-Aldrich) to the supernatant. Phages were collected by centrifugation (16,000 g), the pellet was resuspended in 10 mMTris/1 mM EDTA pH 8.0 buffer (TE) and phages were purified by ultracentrifugation (240,500 g) on cesium chloride gradient (0.5 mg/mL; Sigma-Aldrich). The resulting virions were dialyzed against phosphate buffered saline (PBS) 1X and the concentration of bacteriophage was determined using a spectrophotometer. The spectrum of filamentous fd phages typically exhibits a broad plateau at 260–280 nm with a shallow maximum around 269 nm. Concentration is calculated according to the formula:

mg of phages/mL = (A_269_ – A_320_)/3.84, (1)

assuming that an OD of 1 is equivalent to a concentration of 3.8 mg/mL. fdOVA is a hybrid bacteriophage in which recombinant copies of the major coat protein pVIII are interspersed with wild-type pVIII copies on the coat surface of each single virion. The number of copies of pVIII displaying the OVA (257–264) peptide were estimated based on the relative yields of the various N-terminal sequences obtained by N-terminal sequence analysis of the purified virions, which resulted in 15–20% for each bacteriophage preparation.

### 2.2. Fluorescent Labeling of Bacteriophage Virions

**Figure 1:**
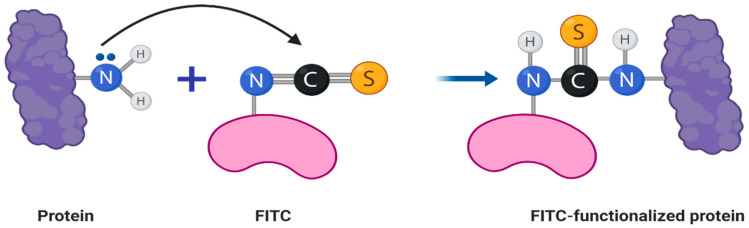
FITC conjugation occurs through the free amino groups of proteins or peptides of the phage scaffold, forming a stable thiourea bond.

Hybrid fdOVA (100 µL) virions (7 µg/µL) in 100 mM carbonate buffer pH 8.2 were treated with a 20-fold molar excess of Fluorescein isothiocyanate (FITC) (Sigma-Aldrich) and stirred gently for 2 h at room temperature (Figure 1). The unreacted FITC was removed with five washes using a 3-kDa MWCO Vivaspinsystem and the bacteriophage was re-equilibrated in PBS 1X pH 7.2 [33]. The concentration of bacteriophage after conjugation was evaluated by UV, as described above.

### 2.3. PLGA Empty MPs

PLGA MPs were prepared by the double-emulsion (W_1_/O/W_2_) solvent evaporation technique [34,35,36]. Briefly, 100 mg poly (lactic-co-glycolic acid) 50:50 (PLGA, RESOMER R RG 504H, 38,000–54,000 Dalton, Boeringer Ingelheim, Kreis, Germany) was dissolved in 1 mL dichloromethane (DCM, Sigma-Aldrich, Kreis, Germany). Next, 100 µL ammonium bicarbonate (ABC, Sigma-Aldrich, Milan, Italy) (7.5mg in 1 mL water) were added and homogenized at 20,000 rpm for 30 s, then mixed with 10 mL of 2% poly (vinyl alcohol) (PVA, Sigma Aldrich, Milan, Italy) and homogenized at 25,000 rpm for 1 min. The final emulsion was added to 40 mL water and stirred to evaporate all the DCM for 3 h. MPs were washed with deionized water three times to remove the PVA then exposed to the lyophilization process overnight.

### 2.4. Bacteriophage Loading on Empty MPs

**Figure 2:**
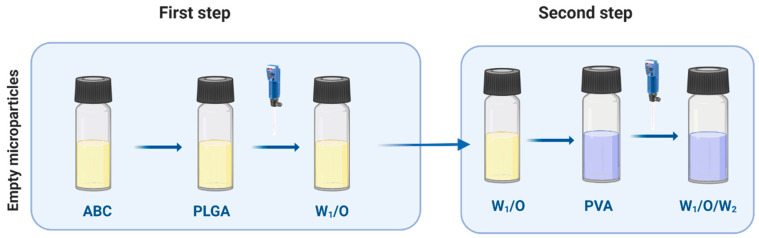
Double-emulsion solvent evaporation method (W_1_/O/W_2_). Fabrication of empty microparticles (MPs).

Empty MPs (5 mg) were suspended in a solution of bacteriophage mixture (0.1 µg/µL) overnight with mild shaking at 4 °C. MPs were then washed three times with PBS 1X and lyophilized (Figure 2).

### 2.5. Particle Size

The size distribution of the MPs was characterized using a laser diffraction instrument (Mastersizer 2000, Malvern Instruments, Malvern, UK). Mastersizer 2000 consists of a 2-mW He–Ne laser (*λ* = 632.8 nm) as the light source, an optic lens and photo-sensitive detectors. lyophilized MPs (3 mg) were suspended in water and added to the tank to measure the particle size distribution in the sample.

### 2.6. Scanning Electron Microscopy of MPs

For scanning electron microscopy (LEO1550), MPs were dispensed on a scanning electron microscopy stub, air-dried for 2 h at room temperature then sputter-coated with 5 nm of gold to make the sample conductive. Imaging was performed with a 5 KeV electron beam.

### 2.7. Confocal Microscopy

Confocal analysis was performed using a Leica SP5 confocal microscope. Fluorescence analysis was achieved using the λ_exc_ at 550 nm, λ_em_ 600–700 for a rhodamine signal and λ_exc_ 488 and λ_em_ 500–600 for the FITC channel. Images were acquired using a HCX IRAPO L 40×/0.95 water objective, a resolution of 1024 × 1024 pixels, zoom 1, and 2.33AU at a maximum pinhole, as already described.

### 2.8. Bacteriophage Release from Particles

Freeze-dried phage-loaded MPs (5 mg) were placed in a 2-mL microcentrifuge tube and suspended in 1 mL of PBS pH 7.4. This mixture was kept under stirring at 50 rpm in a shaker at 37 °C. At defined time points, samples were collected and centrifuged at 3468 g (MICROCL 21R Centrifuge, Thermoscientific, USA) for 5 min. Aliquots of 1 mL supernatant containing phages were taken and replaced with an equal volume of fresh PBS. The supernatants were centrifuged at 9632 g for 10 min. The amount of bacteriophages in the collected supernatant was measured by UV at 269 nm, which is the typical UV signal of phages. All release tests were performed in triplicate over 8 h. The percentage of released phages were related to the total amount of encapsulated phages inside MPs, and the cumulative release was obtained by adding the quantity of phages released at different times.

### 2.9. Encapsulation Efficacy

Freeze-dried PLGA MPs (5 mg) were placed in a 2-mL microcentrifuge tube and dissolved in 375 µL DMSO, then kept in a shaker for 1 h. Next, 150 µL NaOH, 375 µL SDS and 600 µL H_2_O were added. Bacteriophage content was read by UV spectroscopy. Encapsulation efficiency and loading efficiency were calculated using the following formula [29]:

(2) $$\mathrm{Encapsulation}\;\mathrm{efficiency}\;(\%)=\frac{Amount\;of\;phage\;entrapped}{Initial\;amount\;of\;phage}\times 100$$

(3) $$\mathrm{Loading}\;\mathrm{efficiency}\;(\%)=\frac{Mass\;of\;phage\;encapsulated\;by\;microparticles\;}{Mass\;of\;microparticles}\times 100$$

### 2.10. Circular Dichroism

CD spectra were recorded on a Jasco J-1000 spectropolarimeter (JASCO Corp, Milan, Italy), as already reported [37,38]. Spectra were obtained in 10 mM phosphate buffer pH 7.4, blanks (buffer spectrum) were subtracted and standard bacteriophages were analyzed at 0.14 mg/mL.

### 2.11. Colony-Forming Unit Determination

Being non-lytic, filamentous bacteriophages form only transient plaques, thus we used a colony-forming unit (CFU) assay instead of the standard plaque assay to enumerate them [39]. We used fdOVA, a derivative of the engineered fdAMPLAY88 bacteriophage [20] that carries the gene encoding for beta-lactamase (which confers resistance to ampicillin). Infected bacteria were plated on agar plates containing the selective antibiotic to determine phage infectivity. Serial 1:10 dilutions of the bacteriophage were made in PBS 1X and 10 μL of each dilution was added to 200 μL of A_600_ = 0.6 OD TG1*recO* bacterial culture. Each mixture was incubated for 20 min at 37 °C and plated on top of selective LB-agar plates containing 100 ug/mL Ampicillin. A bacterial culture without bacteriophage and a bacterial culture with a known concentration of purified fd bacteriophage were prepared in the same manner as negative and positive controls. Following overnight incubation at 37 °C, the number of colonies was counted for each dilution, and this was used to calculate the number of CFU/mL. Each measurement was performed in triplicate and each experiment was repeated at least three times.

### 2.12. Bone Marrow Derived-Dendritic Cells Generation

Eight- and nine-week-old female C57BL/6 mice were purchased from Charles River (Lecco, Italy) and housed in the IGB “A. Buzzati-Traverso” Animal House Facility under standard pathogen-free conditions abiding by institutional guidelines. Bone marrow-derived dendritic cells (BM-DCs) were produced from precursors isolated from the tibiae of euthanized C57BL/6 mice. Both ends of tibiae were cut and bone marrow was flushed with the needle of a syringe filled with ice-cold RPMI 1640 medium (Invitrogen). Clusters of cells were dissolved by pipetting, and cells were washed twice with medium, plated and cultured with 200 U/mL recombinant murine granulocyte/macrophage colony-stimulating factor (GM-CSF, Peprotech, NJ, USA) in RPMI 1640 medium supplemented with 10% fetal calf serum (FCS), 60 µg/mL penicillin, 100 µg/mL streptomycin, 1 mM sodium pyruvate and 50 µM 2-mercaptoethanol. Immature DCs were collected at day seven of culture and were assayed for dendritic cell phenotypes by staining with the monoclonal antibody anti-CD11c-PE-Cy7 (HL3, BD Biosciences) for FACS analysis.

### 2.13. DC Presentation Assay

**Figure 3:**
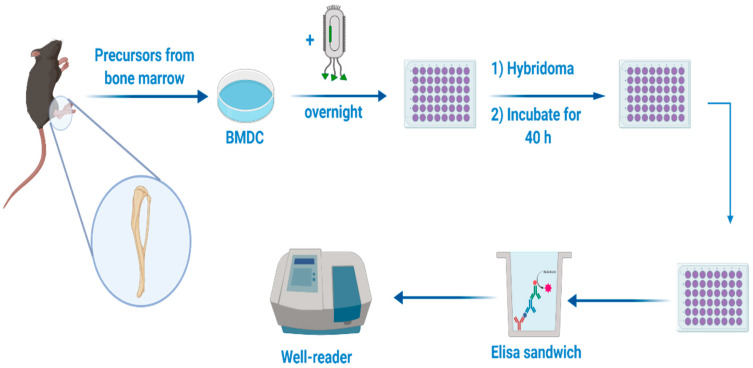
Dendritic cells (DCs) were incubated with bacteriophage overnight. Later, B3Z hybridoma cells were added, and cells were co-cultured for 40 h. Sandwich ELISA was conducted to evaluate Interleukin-2 in supernatants.

1 × 10^6^/mL BM-DCs were incubated overnight with different concentrations (from 0.6 to 6 ug/mL) of free fdOVA filamentous bacteriophage or fdOVA released from MPs. As a control, BM-DCs incubated only with the medium were used. After the incubation, cells were washed twice to remove excess bacteriophages, then co-cultured (100,000/well) with the OTI hybridoma cell line B3Z (50,000/well) for 40 h. B3Z OTI hybridoma cell line, recognizing the OVA (257–264) SIINFEKL determinant, was grown in complete RPMI1640 (10% FCS, 100 U/mL penicillin, 100 μg/mL streptomycin 1% Glutamine, 1% NEM, 1% Sodium Pyruvate, 50 µM 2-Mercaptoethanol). Recognition of the major histocompatibility complex (MHC I)-presented OVA peptide 257–264 (SIINFEKL) by B3Z T cell receptor led to the transcriptional activation of the IL-2 promoter element, resulting in production of IL-2, which correlates with the uptake and processing of the fdOVA and the presentation of OVA (257–264) peptide in MHC I. The amount of IL-2 released into cell co-culture supernatants was measured by ELISA. Supernatants of co-cultures (0.1 mL/well) were assayed in duplicate using mouse IL-2 ELISA MAX Standard (Biolegend, San Diego, CA), according to the manufacturer’s instructions (Figure 3).

## 3. Results

### 3.1. Bacteriophage-Encapsulated MPs

**Figure 4:**
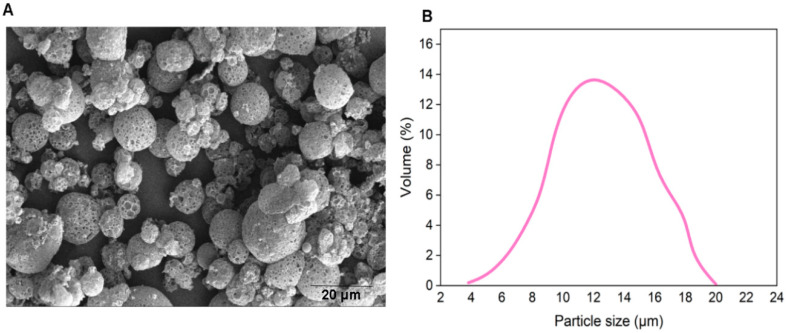
(**A**) SEM of empty MPs. (**B**) Size distribution of porous empty MPs.

We prepared empty porous MPs of PLGA via water-oil-water double emulsion. Porosities were generated by using ammonium bicarbonate (ABC) in the inner aqueous phase. We generated highly porous empty MPs with an average size of d = 14 µm (Figure 4). In this encapsulation method, filamentous bacteriophages were loaded on the surface of generated empty MPs via incubation at 4 °C overnight.

### 3.2. Fluorescent Labeling of Bacteriophage Virions

**Figure 5:**
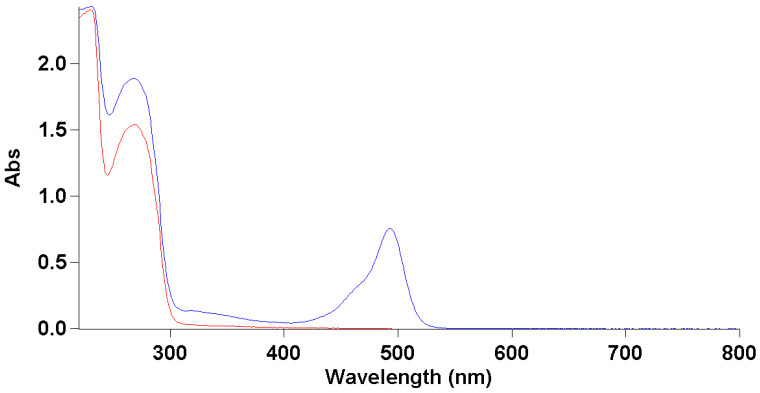
UV spectrum of protein fd bacteriophage before and after conjugation with FITC. The red line is the UV spectrum of bacteriophage before conjugating with FITC. The blue line is the UV spectrum of bacteriophage after conjugation with FITC.

To study the correct encapsulations of bacteriophage inside MPs, we functionalized them with the FITC dye as reported in Section 2. The correct conjugation was evaluated by UV-Vis, which showed the presence of two shoulders—one at 490 nm typical of the dye, and one at 280 nm that was related to the proteins of the bacteriophage coat (Figure 5). We also obtained a good degree of loading (DOL) equivalent at 0.7. Thanks to this result, we performed a deeper morphological characterization of bacteriophage-loaded MPs by confocal microscopy.

### 3.3. Confocal Characterization of Bacteriophage-Loaded MPs

**Figure 6:**
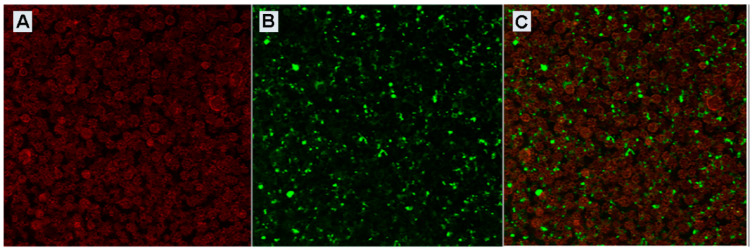
UV spectrum fd-OVA and fd-OVA FITC. (**A**) Rhodamine-loaded-MP confocal microscopy analysis. Rhodamine channel (λ_exc_ 550, λ_em_ 600–700). (**B**) FITC channel (λ_exc_ 488, λ_em_ 500–600). (**C**) Overlay of the channels.

In detail, bacteriophage-loaded PLGA MPs were synthesized to see the PLGA structure using an excitation source at 550 nm and an emission range of 600–700 nm. Moving the λ_exc_ to 488 nm and the emission band between 500–600 nm, we were able to evaluate the correct bacteriophage deposition within an external shell of the MPs against the post-encapsulation method and this was also confirmed by the overlay of the two channels (Figure 6).

### 3.4. In Vitro Controlled Release

**Figure 7:**
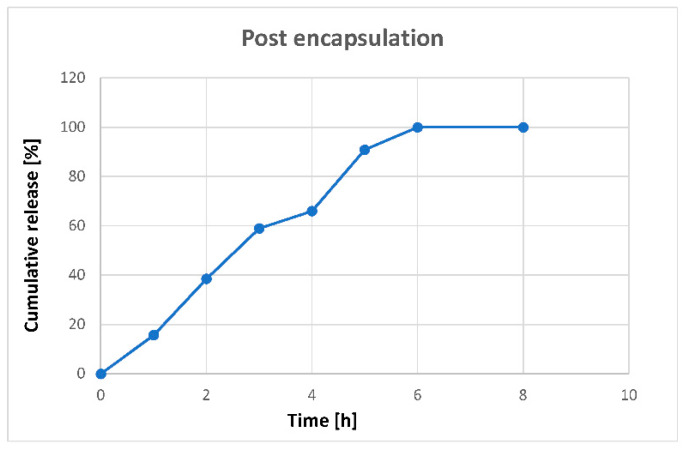
Release of bacteriophage from MPs. Bacteriophage release was measured over time after suspending the MPs in PBS.

The release of bacteriophages was evaluated for post-encapsulated bacteriophage-MPs over time. After resuspension of the MPs, bacteriophage started suddenly to be slowly released. After 6–8 h, practically all bacteriophages had been released (Figure 7).

### 3.5. Encapsulation Efficiency

Bacteriophage content was evaluated by UV-Vis spectroscopy following its characteristic peak at 269 nm, and concentration was evaluated by the Beer–Lambert law. Encapsulation efficiency and loading efficiency were 40% and 0.8%, respectively.

### 3.6. Circular Dichroism

**Figure 8:**
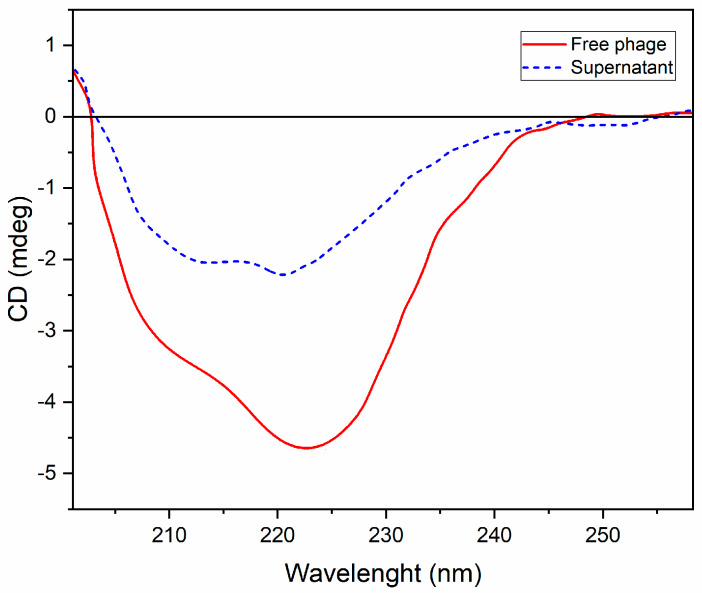
A Circular dichroism (CD) spectrum of free bacteriophage (red spectrum) and bacteriophage released from post-encapsulated MPs (blue spectrum).

Circular dichroism (CD) analysis of free bacteriophage was compared to the bacteriophage encapsulated within MPs, then released in phosphate buffer pH 7.4. In particular, as reported in Figure 8, the free bacteriophage showed a mixed α-helix–β-sheet conformation at a concentration of 0.35 mg/mL (blue spectrum), with a pronounced minimum at 222 nm (typical of the β structures) and two shoulders: one negative at 205 nm, and one positive at 190 nm, which are characteristic signs of ellipticity. The same considerations can be made for the bacteriophage released from the post-encapsulated MPs (6 h, 0.14 mg/mL (red spectrum)), which showed the mixed α-helix–β-sheet conformation to be a free underlying bacteriophage, since this incubation methodology does not affect protein stability.

### 3.7. Biological Activity of Filamentous Bacteriophage after Encapsulation

To verify the structural and functional integrity of the bacteriophages and their biological activity after lyophilization and encapsulation in MPs, particles containing post-encapsulated virions were reconstituted in PBS and allowed to spontaneously release the bacteriophage MPs in the supernatant. The MPs were removed by centrifugation, then the bacteriophage-containing supernatant was used to infect TG1 *E. coli* bacterial cells to determine the bacteriophage titre. The filamentous fdOVA bacteriophage used was a derivative of the engineered fdAMPLAY88 bacteriophage [20], which carries the gene encoding for beta-lactamase and confers resistance to Ampicillin. Only bacteriophage-infected bacteria can grow on ampicillin-containing plates, forming colonies.

**Figure 9:**
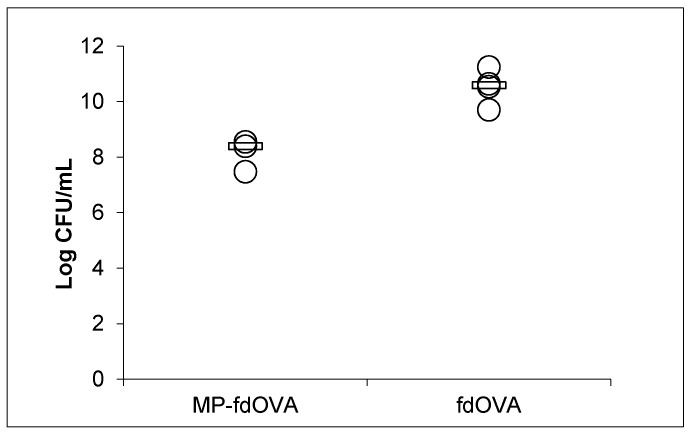
Effect of the encapsulation process on the biological activity of the bacteriophage. Infectivity of free filamentous bacteriophage fdOVA or fdOVA released from MPs (MP-fdOVA). The infectivity was expressed as the number of colony-forming units of TG1 *E. coli* bacterial cells infected with bacteriophage that were able to grow on Ampicillin plates. Each measurement was performed in triplicate, and the median of three different experiments is reported (black line). Differences are not significant (*p* > 0.05 by Student’s *t*-test).

Bacteriophage released after post-encapsulation was able to infect bacteria with only a slight loss of titre compared to the free fdOVA bacteriophage, suggesting that bacteriophages were still intact, capable of infecting bacteria and viable, even after being encapsulated and released by PLGA MPs (Figure 9).

### 3.8. Antigen-Specific Immune Response to Encapsulated fdOVA Bacteriophage

**Figure 10:**
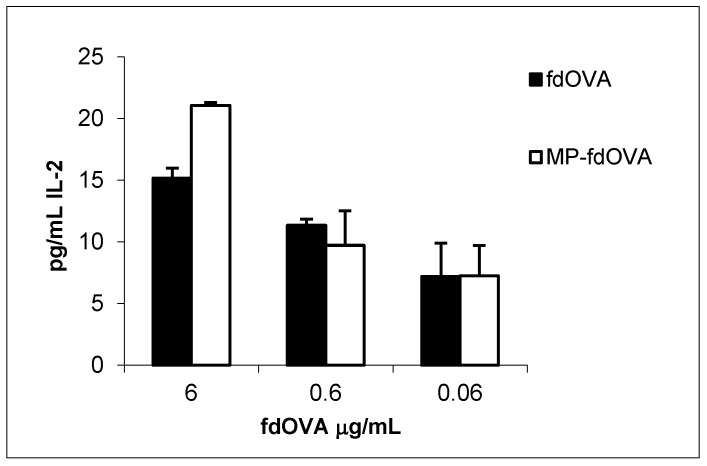
IL-2 release of B3Z hybridoma cell line in response to fdOVA delivering OVA (257–264) SIINFEKL peptide. Bone marrow-derived dendritic cells (BM-DCs) were incubated with graded doses of fdOVA bacteriophage free or released from post-encapsulated MPs. BM-DCs were co-cultured with B3Z hybridoma cells for 40 h and supernatants were assayed in duplicate. Mean + SD is reported, and one representative experiment (of two) is shown. Differences are not significant by Student’s *t*-test (*p* > 0.05).

We evaluated the immunological response to the antigen delivered by bacteriophage particles after the encapsulation process. Antigen presentation functions are crucial for the activation of an immune response. Mouse BM-DCs were incubated overnight with free fdOVA bacteriophage or fdOVA bacteriophage prior to release from MPs. DCs were then assessed for their capability to process filamentous bacteriophage and activate OVA (257–264)-specific hybridoma T cell B3Z, a CD8+ T cell line known to produce IL-2 exclusively upon activation of a T cell receptor specific to the OVA (257–264) SIINFEKL peptide in the context of H-2Kb MHC class I complex, expressed on APCs. B3Z activation in response to OVA (257–264) was measured by interleukin-2 (IL-2) released in the culture supernatants. We found that fdOVA bacteriophage released from MPs after post encapsulation was able to be taken and processed by dendritic cells, inducing a B3Z response similar to the one obtained using free fdOVA bacteriophage (Figure 10). Empty MPs used at the highest concentration did not induce IL-2 release (data not shown).

## 4. Discussion

We have demonstrated the ability of filamentous bacteriophage-carrying immunogenic peptides to be encapsulated in PLGA microparticles. We assessed that recombinant fd bacteriophages, lyophilized after encapsulation in MPs and reconstituted, are released from MPs maintaining their immunogenic properties intact, which demonstrates the use of fd phage-MPs as nanocarriers for antigen delivery and as efficient triggers of specific immune responses. It should be noted that the CFU assay described allows the enumeration of the infective bacteriophages. Our results demonstrated that encapsulation of bacteriophage into PLGA MPs via emulsification and lyophilization results in biologically active bacteriophages, preserving the integrity of the proteins of the coat.

Up to now, the encapsulation of other strains of bacteriophage in nano/microsystems has been successfully achieved, especially for bacteriophage therapy purposes. For instance, lipid cationic mixture [40] and a combination of alginate with the antacid CaCO3 [41] were used for encapsulation of Salmonella-specific bacteriophage, whereas PLGA microspheres were used to encapsulate selective bacteriophages for *Staphylococcus aureus* or *Pseudomonas aeruginosa* [42]. However, to the best of our knowledge, this is the first report of the encapsulation and delivery of recombinant fd bacteriophage particles by PLGA microparticles for immunotherapeutic purposes.

PLGA is a versatile polymer that is widely used for the microencapsulation of therapeutic drugs, proteins and nucleic acids [43,44]. Thanks to its biodegradable nature and biocompatibility characteristics, it is an excellent vehicle for the delivery of vaccine formulations and has recently been considered safe by the US Food and Drug Administration and the European Medicine Agency.

To be sure about the maintenance of bacteriophage stability upon encapsulation, we developed a post-encapsulation method. The main advantage is that the encapsulation of bacteriophage in an inert and biodegradable polymer can protect bacteriophage from degradation due to proteases or harsh environments, while allowing easier storage conditions. Additionally, the biodegradable nature of the PLGA permits a prolonged bacteriophage release, providing improved pharmacokinetic [45]. The sustained release of drugs or therapeutics offers considerable advantages, allowing decreased numbers of administrations and reduced pharmacotoxicity, due to accumulation in the blood and regulation of the drug release. Furthermore, the DNA of bacteriophage has a CpG motif, meaning that there is no need to add any exogenous adjuvant to the formulation of the vaccine [18]. Since fd bacteriophage is non-pathogenic and non-lytic for eukaryotic cells, its use has immense promise for humans in the coming years. This intrinsic advantage of bacteriophage was translated to the fd-encapsulated PLGA MPs, confirming immunization without utilizing exogenous adjuvants.

Our results indicate that PLGA MPs can also preserve the structure and viability of bacteriophage after the lyophilization process, allowing a gradual release after resuspension and an effective delivery of the payload to DCs. Thus, bacteriophage is well-suited for antigen delivery, due to inexpensive production in large quantities, safety and potent adjuvant capacities. In addition, the possibility to maintain bacteriophages encapsulated in MPs in a lyophilized form could lengthen the storage time of the encapsulated vaccine. Overall, this property may represent a great practical and economic advantage for the storage and administration of phage nanoparticles in underdeveloped geographical areas.

Functionalization of the PLGA polymer (e.g., via PEGylation) could also allow binding on the microparticle surfaces of antibodies for targeted delivery in body tissues, or could lead to immunostimulating molecules to enhance immune response to vaccines [46,47,48]. Various molecules have been associated with PLGA-based particle surfaces including peptides, proteins, carbohydrates, nucleic acids, aptamers and other small molecules such as folate, anisamide and phenylboronic acid. These ligands can be either directly conjugated or adsorbed onto the surface of assembled MPs, or they can be associated with one of the polymer components during assembly [49]. Encapsulating a phage-based vaccine in MPs decorated with antibodies for targeting specific cell subpopulations, or delivering cytokines or growth factors to stimulate immune responses, could be valuable tools in generating multifunctional PLGA particles that combine different approaches for biomedical applications.

In summary, we present a fabrication method for encapsulating fd filamentous bacteriophage into PLGA polymeric MPs that grants both bacteriophage stability and infectivity, as well as the immunogenicity of the antigen displayed on bacteriophage.
